# Evaluating Depression Care Management in a Community Setting: Main Outcomes for a Medicaid HMO Population with Multiple Medical and Psychiatric Comorbidities

**DOI:** 10.1155/2012/769298

**Published:** 2012-10-22

**Authors:** Jeanette A. Waxmonsky, Marshall Thomas, Alexis Giese, Steve Zyzanski, L. Miriam Dickinson, Gretchen Flanders McGinnis, Paul Nutting

**Affiliations:** ^1^University of Colorado Denver (UCD) Depression Center and Department of Psychiatry, University of Colorado School of Medicine, Building 500, 13001 E. 17th Place, Aurora, CO 80045, USA; ^2^Colorado Access, 10065 E. Harvard Avenue, Suite 600, Denver, CO 80045, USA; ^3^Case Western Reserve University, Bolwell 1200, 11100 Euclid Avenue, Cleveland, OH 44106, USA; ^4^Department of Family Medicine, University of Colorado Denver, Academic Office 1, 12631 E. 17th Avenue, Aurora, CO 80045, USA; ^5^Center for Research Strategies, 225 E. 16th Avenue, Suite 1150, Denver, CO 80203-1694, USA

## Abstract

The authors describe the implementation of a depression care management (DCM) program at Colorado Access, a public sector health plan, and describe the program's clinical and system outcomes for members with chronic medical conditions. High medical risk, high cost Medicaid health plan members were identified and systematically screened for depression. A total of 370 members enrolled in the DCM program. Longitudinal analyses revealed significantly reduced depression severity scores at 3, 6, and 12 months after intervention as compared to baseline depression scores. At 12 months, 56% of enrollees in the DCM program had either a 50% reduction in PHQ-9 scores or a PHQ-9 score < 10. Longitudinal economic analyses comparing 12 months before and after intervention revealed a significant but modest increase in ER visits, outpatient office visits, and overall medical and pharmacy costs when adjusted for months enrolled in DCM. Limitations and recommendations for the integrated depression care management are discussed.

## 1. Introduction

In 2004, Colorado Access, a nonprofit public sector health plan, developed and implemented an internal depression care management program to improve depression treatment outcomes, increase appropriate utilization of medical services, and reduce health care costs. In order to achieve these goals, Colorado Access developed a risk-stratification algorithm to identify a target population of high cost health plan members with depression and chronic medical illnesses who were likely to benefit from care management through a proactive care management plan delivered by care managers [1]. This paper describes the care management program and its outcomes, challenges in measuring outcomes for Medicaid populations, and future directions for integrated behavioral and medical care management services.

Over the past decade, collaborative care models for depression and chronic medical diseases have demonstrated success in population-based case finding and employment of evidence-based treatments for depression [2]. Meta analyses of such studies have yielded moderate effect sizes in reducing depressive symptoms as well as improving medical illness outcomes [3, 4]. 

As part of the Robert Wood Johnson Foundation's initiative on aligning clinical and economic systems to sustain depression care management for primary care, Colorado Access combined depression care management with economic and nonfinancial incentives to reorganize systems of care for depression treatment [5]. Colorado Access utilized the Chronic Care Model's (CCM) clinical framework to develop and integrate depression care management into its existing health plan infrastructure and support depression treatment in primary care [6, 7]. 

Unlike many depression care management programs that are practice based, Colorado Access designed its program to be operated at the health plan level in order to create efficiencies and consistency in program delivery and to be minimally burdensome to primary care providers, that is, having little impact on existing provider clinical practices [1]. The program utilized a multifaceted depression intervention that involved changes within the CCM health care components: leadership, delivery system design, clinical information systems, decision support, self-management support, and community resources and health care policies [7]. Evidence suggests that CCM multicomponent interventions are effective in promoting informed members who take an active part in their care and more effective providers with enhanced resources and expertise [8]. Also, data from randomized trials demonstrate that multifaceted interventions are more likely to improve depression outcomes than single component interventions [8–10]. 

A prior pilot demonstration of depression care management along with cost analyses of health plan members with medical and psychiatric comorbidities helped create the business case for the Colorado Access Board of Directors and executive leadership to support program implementation on a larger scale [1, 11]. Within the Colorado Access health plan, the existing intensive care management services delivery system was redesigned to incorporate the depression care management intervention. 

## 2. Methods

### 2.1. Identification of Targeted Care Management Population

The majority of depression care management members were identified through a risk stratification method that identified high risk, high cost health plan members. These members had chronic, often multiple, medical illnesses (e.g., diabetes, congestive heart failure) and were at risk for high future health care costs as evidenced by Chronic Disability Payment System scores at the 90th percentile [12]. Based on the prior cost analyses, it was predicted that improved management of both depression and other medical conditions would lead to improved clinical and economic outcomes [11]. 

In addition to identifying cases via the risk stratification method, Colorado Access providers were able to directly refer their members with depression and comorbid chronic medical illnesses to the program; care management staff screened these members to determine whether they met criteria for enrollment. A third subset of high cost, high risk members with diabetes was identified at the Pueblo Community Health Center (PCHC) clinics in Pueblo, Colorado. Funding from the Caring for Colorado Foundation allowed for the development of a hybrid model of depression care management, in which a care manager was located on site at the PCHC clinics but utilized the same electronic registry, screening tools, protocol, care management educational material, and supervision process as the plan-based Colorado Access care managers. 

### 2.2. Care Management Staff and Training

 Registered nurses and health-plan-based consumer navigators were trained in depression care management to complement existing care coordination services to members with medical illnesses. Consumer navigators worked with the nurse care managers in providing additional psychosocial support and connecting health plan members to community resources. The standardized training included the depression care management intervention and protocols, evidence-based treatments for depression, member education and self-management materials, and community resources. Additionally, care management staff received training on motivational interviewing for persons with chronic medical illnesses and depression, developing care plans, and prioritizing treatment goals. 

### 2.3. Clinical Information Systems and Care Management Registry

The health plan information system was systematically analyzed for administrative and claims data to identify high medical risk, high cost health plan members with depression who were likely to benefit from more intensive care management support. Also, care managers were given access to pharmacy, lab, inpatient, and emergency department reports, which assisted in the development of care plans and care coordination strategies. Structured assessment, care plans, care manager followup, and supervision notes were tracked in an electronic health plan registry to aid clinical processes (e.g., timing of follow-up contacts and outcomes tracking). The electronic registry software was internally developed by the Colorado Access health plan and included care manager screens and assessments, individualized care plans, and salient clinical information. The registry helped facilitate supervision and feedback as well as monitor care manager performance (e.g., generate caseload reports) and track clinical outcomes.

### 2.4. Mental and Physical Health Screening and Intervention

The identified cohort of health plan members was screened telephonically by care managers using a health screening questionnaire that included the Patient Health Questionnaire-9 item depression screen (PHQ-9; [13]). Members with PHQ-9 scores of 10 or above were enrolled into the depression care management program. Additional screens for other psychiatric conditions (dysthymia, anxiety, psychosis, bipolar disorder, and substance abuse), physical health conditions, and psychosocial needs were also conducted during enrollment. Care managers developed a prioritized care management plan utilizing this screening information plus administrative and medical utilization data. Care plans included the domains of medical care self-management, community involvement, and social support. An objective (a measurable treatment goal) and an intervention were established for each domain. Text fields under each identified goal allowed for input of individual member notes/report, care management notes, supervision notes, next steps, and goal resolution reason.

### 2.5. Supervision

Supervision of care managers was provided by the plan's medical director, a psychiatrist, and a psychologist on a weekly basis using a case conference format. The supervision team reviewed the care management plan, assisted with formulating and prioritizing complex needs, and defined achievable goals. The supervision team also helped the care manager design individually tailored member education and self-management goals for depression and medical illnesses. Additionally, linkages to community resources for depression needs and other services were enhanced by the interaction of medical and mental health staff the supervision meeting. The supervision team also provided education and consultation to health care providers.

The depression care management program used a stepped collaborative care approach, in which the supervision process helped identify those members whose depression could be appropriately treated in a primary care setting and those members with comorbid psychiatric diagnoses who required specialty behavioral health services. Additionally, through careful monitoring of depression symptoms and treatment response, the supervision process allowed for identification of members with treatment resistant depression who needed specialized behavioral health consultation or treatment. 

### 2.6. Member Followup

Members enrolled in the DCM program received a monthly follow-up call to determine whether they had started or continued depression treatment, were making progress towards their individualized self-management goals, were experiencing any obstacles, and to assess their current level of depression using the PHQ-9. A score of 5 or less on the PHQ-9 for 3 months or longer was used as criteria for successful remission from depression. An effort was made to follow all members enrolled in the DCM for a minimum of one year (up to two years maximum) regardless of their depression remission rates.

### 2.7. Illness Self-Management and Patient Resources

 Colorado Access tailored illness management strategies for enrolled program members based on assessments of barriers to accessing care, understanding of specific illnesses (e.g., depression, diabetes), readiness for behavioral change (motivational interviewing/stage of change), and educational materials. Depression self-management and educational materials were modified from the MacArthur Reengineering Systems for Primary Care Treatment of Depression Program (RESPECT-Depression; [6]) and were available in English and Spanish. Colorado Access developed a community resource book with resources for depression treatment, as well as for transportation, additional services, and support groups that care managers used to assist enrolled program members.

### 2.8. Identification of the Evaluation Cohort

**Figure 1:**
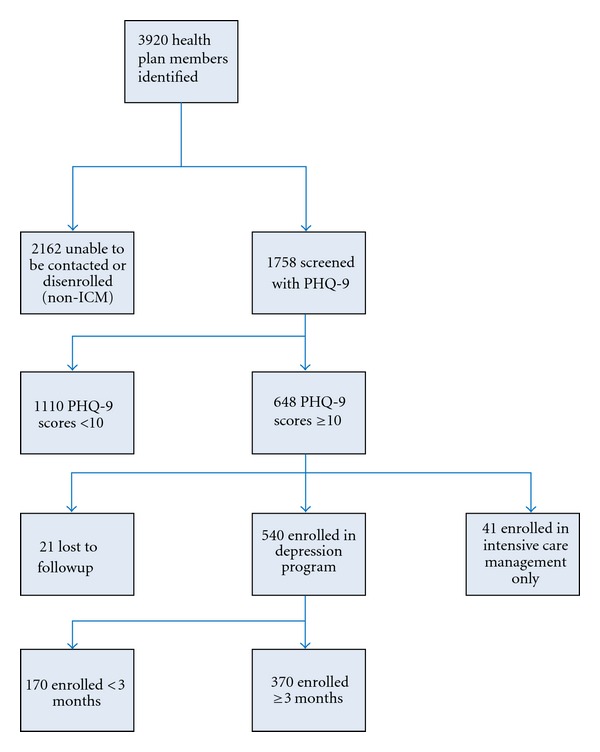
Health plan member evaluation cohort.

A total of 3,920 adult Medicaid health plan members were identified through the Colorado Access risk stratification algorithm as high risk, high cost members (see Figure 1). Of this group, 2,162 were unreachable by phone or were no longer enrolled in the health plan at the time of screening. The remaining 1,758 members were screened for depression using the PHQ-9; 648 (36.9%) had an initial PHQ-9 score of 10 or above (indicating a positive screen for depression) and were offered participation in the DCM program. A total of 540 agreed to participate and had at least one care management contact (83% enrollment rate). Members enrolled in the DCM program received at least monthly depression assessment and care plan review phone calls and occasional in-person contacts by care managers for up to two years. Of the 540 members enrolled, 370 members (68.5%) were enrolled for at least 3 months; 170 members (57.1%) were enrolled for 3 months or less. Persons in DCM dropped out for the following reasons: unable to be further reached by telephone (*n* = 85), loss of Medicaid eligibility (*n* = 74), and no longer interested in care management (*n* = 11).

### 2.9. Statistical Analyses

Descriptive statistics were calculated to characterize demographic variables, medical and psychiatric comorbidities, and length of time enrolled in the depression care management program. The primary outcome measures included depression severity as measured by the PHQ-9 < 10 and 50% reduction in depression severity as measured by the PHQ-9 for response rates. Additionally, telephone satisfaction surveys were conducted for a small subgroup of providers (*n* = 20) and health plan members (*n* = 81) enrolled in the depression care management program at 12 months after program implementation. 

General linear mixed effects models (random intercept, random slope) were used to analyze trajectories of depression scores over time for members enrolled in the depression care management program [14–16]. Repeated measures within members were modeled as a linear trend growth curve model with time coded as days since baseline PHQ-9 score and converted to month for ease of interpretation; quadratic trend was tested but did not significantly improve fit. Covariates were included if they were significantly associated with depression scores or were associated with length of follow-up interval. Potential moderators of improvement in PHQ-9 scores were tested one at a time by adding two-way interactions to the model. All analyses were conducted with SAS Version 9.3 [17].

### 2.10. Cost and Utilization Analyses

Cost and utilization data for 12 months prior to enrollment and 12 months after enrollment were estimated for 269 patients with utilization data for both periods. If patients were enrolled for less than a full 12 months, cost estimates were adjusted to reflect the 12-month before or after period prior to analysis. Before and after costs were analyzed using generalized linear mixed effects models (Poisson for counts, gamma distribution for costs) in SAS V9.3 [17, 18] to determine whether utilization differed between the two time periods. 

## 3. Results

### 3.1. Depression Treatment Outcomes

The final depression care management evaluation cohort (370 health plan members who were enrolled in the intervention for 3 months or more) had a mean age of 58 years (range 22–88 years old) and were 81% female and 45% Caucasian. 

Linear growth curve models with sociodemographic and clinical covariates were used to evaluate change in depression symptoms over time (see Table 2). DCM members improved at a rate of approximately 0.6 reduction per month in PHQ-9 scores per month (*F*(1,110) = 195.74, *P* < .0001). Longitudinal analyses were adjusted for gender, age, race/ethnicity, marital status, non-English language, bipolar disorder, psychotic disorder, anxiety, diabetes, cardiac disease, and pulmonary disease (See Table 2). At baseline, the following variables were associated with worse baseline PHQ-9 scores: marital status (*P* = .02), non-English language (*P* = .04), and having a positive bipolar disorder screen (*P* < .0001). Longitudinally, there was evidence of differential intervention effects by non-English language (*P* = .04) and anxiety (*P* = .04), with non-English speakers showing more improvement in PHQ-9 scores over time and patients with a positive anxiety screen showing less improvement in PHQ-9 scores over time.

For members enrolled in the depression care management program at 3 months (*n* = 269), 45% had a 50% reduction in PHQ-9 scores, 55.8% achieved a PHQ-9 score below 10, and 57.6% had either a 50% reduction in PHQ-9 scores or achieved a PHQ-9 score below 10 (see Table 3). For members enrolled in the depression care management program at 6 months (*n* = 197), 48.7% had a 50% reduction in PHQ-9 scores, 56.9% achieved a PHQ-9 score below 10, and 58.4% had either a 50% reduction in PHQ-9 scores or achieved a PHQ-9 score below 10. At 12 months, (*n* = 84), 45.2% had a 50% reduction in PHQ-9 scores, 54.8% achieved a PHQ-9 score below 10, and 56.0% had either a 50% reduction in PHQ-9 scores or achieved a PHQ-9 score below 10.

As noted in Table 1 above, psychiatric comorbidities were prevalent among the depression care management members. Over 51% had dysthymia in addition to a current major depression episode (*n* = 190), and 33.2% screened positively for bipolar disorder (*n* = 123). Only 31.4% of the sample (*n* = 114) did not screen positively for a comorbid psychiatric disorder, and it was this group with major depression only that demonstrated the greatest improvement in PHQ-9 depression severity scores. 

### 3.2. Medical Utilization and Cost Outcomes

Medical utilization and costs were calculated at 12-month before and after intervention for the DCM members (see Table 4). During the 24-month time period, there was an increase in ER visits (from 0.84 to 1.57 average ER visits per member), outpatient office visits (1.42 to 4.34 average outpatient office visits), and net pharmacy costs ($3528 to $4655) all with *P* values < .01. As a result of increased ER, outpatient visits, and net pharmacy costs, the average net medical and pharmacy costs rose from $11,676 to $13,300 (*P* < .05). 

### 3.3. Provider and Health Plan Member Satisfaction

A baseline survey of a sample of primary care providers (*n* = 12) indicated that about 71% found the care management program helpful to their members and were satisfied with program. 100% of these providers stated that they would refer more members to the program. A follow-up survey conducted 6–8 months later with this sample found that 100% believed that the care management (CM) was helpful in meeting their members' depression needs, 100% believed that their members were benefiting from CM, and 91% were satisfied overall with CM. Providers stated that they found that CM was “helpful with very disorganized and needy members” and that “care manager reassessment of members frequently allowed the provider to know when to make medication adjustments or to add therapy.” A sample of members in the depression CM program (*n* = 39) was also surveyed. Baseline and follow-up telephone satisfaction surveys of the subgroup of members with diabetes and depression enrolled 6 months or longer indicated that 100% of members were either very satisfied or satisfied with the help they received from the care manager. Health plan members stated that CM provided encouragement, understanding, and support, which was helpful with self-management goals and medication refills, provided education about depression and diabetes, and assured follow-up care after hospitalization. 

## 4. Discussion

The clinical and economic outcomes of the Colorado Access integrated depression management program have allowed for the sustainability of this intervention. The DCM program positively impacted depression symptom severity scores over time. Although the program increase health care costs by an average of $1624 per health plan member, most of these costs can be attributed to increased outpatient visits and net pharmacy costs rather than ER admissions or acute hospitalizations. Additionally, health plan members enrolled in the program and their primary care providers were generally positive about the program. Importantly, the depression CM program led to long-term system changes within the health plan that supported the sustainability of the program after grant funding ended. Depression care management has become one of the core competencies of Colorado Access health plan's intensive care management model. 

The outcome results of this program are limited methodologically in several important ways. Health plan member recruitment was low as most Medicaid members were not able to be reached and some members had disenrolled in the health plan by the time of recruitment. There were several ways that members could be identified for enrollment in the program: through the Colorado Access risk stratification methodology, through provider referral, and through identification of high risk, high cost members with depression, and comorbid diabetes at one health center (Pueblo Community Health Center). Thus, there is likely to be selection bias as providers only referred members for which they were having difficulty in managing or coordinating care. However, the group of provider referred health plan members to DCM was a small number. Additionally, there was no randomization to the DCM program. 

 At 6 months, the average PHQ-9 depression severity score was reduced by 37% or an average 5.6 points (from an initial average of 15.1 to 9.5). PHQ-9 change scores of 5 or greater indicate a clinically relevant change in individuals receiving depression treatment [19]. At 6 and 12 months, DCM members had had a successful response rate of 58.4% and 56%, respectively, (as measured by either a 50% reduction in PHQ-9 scores or a PHQ-9 < 10) which suggests that the intervention had an impact on reducing depression scores. These results are consistent with those found for the IMPACT randomized controlled trial and posttrial intervention with older adults which found 6-month reduction rates of 5.6 and 6.3 points, respectively, in PHQ-9 severity scores [20]. It is important to note that, unlike some randomized controlled trials (RCTs) of depression care management (e.g., [21, 22]), the current study cohort was not limited to first episodes of depression, but included persons with recurrent, chronic, and treatment-resistant depression. Also, members who screened positive for psychiatric comorbidities were not excluded in this evaluation. It would be expected that, if the intervention did not affect depression scores, that there would not be a decrease in depression scores similar to those found in RCTs with more homogeneous samples. 

One of the biggest challenges in conducting depression CM with a Medicaid population was contacting health plan members over the study period, as this population is highly mobile. Since most care management contacts occurred telephonically, care managers often had difficulty with reaching program members by telephone. The care managers often had to make multiple call attempts to reach health plan members and would often need to contact primary care clinics to get updated phone numbers. Care managers found that members often lacked consistent phone numbers or addresses and would have to engage primary care clinics and other contacts in order to locate them. 

Many members had multiple, complex medical, psychiatric, and psychosocial issues that would prove challenging for the primary health care providers and the care managers to address. The supervision process addressed this challenge by helping the care managers identify two or three high priority issues and balance these with the member's priorities. For example, sometimes the member's medical conditions or transportation issues needed to be treated first before addressing his or her depression treatment needs and vice versa, sometimes a member's depression needed to be treated before he or she could engage in self-management strategies for controlling diabetes. Care managers also had to learn how to balance what they saw as the member's medical and behavioral health treatment needs with the member's perception of their medical and behavioral health needs. Additionally, it was important that the care manager could identify potential barriers to treatment adherence early in the process. 

### 4.1. Lessons Learned

Staff development was a major step in implementing the depression CM program: staff had to learn to assess and monitor depression, screen for other psychiatric comorbidities, and implement care plans that addressed depression treatment and self-management goals. Some were not interested or able to make this change, and staff turnover was high during the course of the two year project. New care managers were recruited for the program, but this took substantial time for recruiting and training. Recruitment efforts sought out nurses who were comfortable working with health plan members, who had complex psychiatric and medical comorbidities, and/or who had previous experience with members with behavioral health issues.

Another lesson learned was that centralized health plan-based depression care management and provider site-based depression care management have different, distinct advantages and limitations. Providing case finding and care management at the health plan allowed efficient incorporation of the program into existing systems allocation of resources across many provider sites and members. In contrast, delivering care management on site at primary care clinics required substantial start up time because each clinic had its own information systems, clinical processes, and unique culture. Colorado Access had found from its previous experience with a randomized controlled trial of site-based depression care management that this model is resource-intensive in terms of staff training and requires developing unique clinical processes and supervision for each clinic. 

A hybrid model offered some advantages. In this model, a care manager was located on site at one or more primary care practices while utilizing the plan-based clinical information systems and decision support (e.g., electronic registry, screening tools, protocol, supervision). This allowed for face to face contact with members and easier followup. The care manager was also part of the clinic staff and familiar with staff culture, which made it easier to coordinate care on site for members, utilize local medical record systems, and coordinate with other community resources. 

## 5. Conclusions

The depression care management program showed favorable clinical outcomes and high levels of member and provider satisfaction, leading Colorado Access to adopt the program as one of its core competencies and a valuable service for providers and health plan members. In contrast to other collaborative care models for depression treatment in primary care settings, the program did not require significant changes to primary care practices because member identification, engagement, and followup occurred primarily at the plan level [23, 24]. Providers stated that the psychiatry supervision and consultation provided through Colorado Access' care management team was helpful to them in managing member care as well as facilitating referrals to specialty mental health services. 

Colorado Access is continuing its and collaboration with partner providers to develop new care management models and implementation strategies. High volume safety net practices such as the Federally Qualified Health Centers (FQHCs) may be more appropriate for on-site care management models, which fully integrate depression care management into the particular system of care at a given site. This model clearly has advantages, but also potential challenges: differences in the skill sets and day-to-day activities of the care managers at practice sites and variability in training and data collection. One solution may be a hybrid model in which the health plan would hire, train, and provide both on-site and centralized access to data collection tools, member education resources, and a multidisciplinary team of clinicians to provide supervision. In this manner, a standardized care management model could be utilized while accommodating the unique features and needs of each practice. This model would require a contracting platform that allows funding to flow to the primary care site to support care management and delineates how the site-based care managers will remain integrated with the health plan.

## Figures and Tables

**Table 1:** Demographics, comorbidities, and length of participation of DCM members.

Variables	DCM *n* = 370
Age (mean years)	57.9
Range 22 to 88 years	
Months in program (mean)	10.4
Range 3 to 27 months	
Mean baseline PHQ-9 score	15.0
Language	
% English	96.4
Gender	
% Female	80.5
Race	
% White	45
% Hispanic	31
% African-American	10
% Other	4
% Unknown	10
Marital status	
% Divorced	33
% Married	19
% Separated	5
% Single	28
% Widowed	5
% Unknown	10
Comorbidities	
% Bipolar	33.2
% Schizophrenia	5.0
% Anxiety	12.7
% Psychosis	8.9
% Dysthymia	51.4
% Substance Abuse	8.9
% Diabetes	41.1
% CHF	10.8
% CAD	13.8
% COPD	21.9
% Asthma	26.2

**Table 2:** Longitudinal analysis of PHQ-9 scores over time.

	Coefficient	SE	*P* value
Independent variables			
Intercept	10.49	1.60	<.0001
Age	.02	.02	.4908
Race/ethnicity			.8429
Non-Hispanic white (ref) 4	0	—	
African American 3	−.13	.81	
Hispanic 2	−.50	.56	
Other 1	−.29	.91	
Marital status			.0219
Married (ref)	0	—	
Widowed 4	−2.41	1.17	
Separated/divorced 3	.50	.66	
Single 2	−.60	.70	
Unknown 1	−1.62	1.08	
Non-English speaking	2.43	1.16	.0373
Bipolar disorder	2.20	.55	<.0001
Psychotic disorder	.75	.93	.3635
Anxiety	.15	.76	.8465
Diabetes	.62	.50	.2205
Cardiovascular disease	−.07	.62	.9088
Pulmonary disease	.52	.50	.2963
Slope terms			
Change per month in DCM group	−.59	.04	<.0001
Difference in slope for non-English speaking	−.65	.31	.0380
Difference in slope for patients with anxiety	.23	.11	.0418

**Table 3:** Depression response over time as measured by PHQ-9 severity scores.

Response in DCM group				
	50% reduction in PHQ-9 score	PHQ-9 < 10	Either 50% reduction or PHQ-9 < 10	Total *N*
	% (*N*)	% (*N*)	% (*N*)	
3 months	45.0% (121)	55.8% (150)	57.6% (155)	269
6 months	48.7% (96)	56.9% (112)	58.4% (115)	197
12 months	45.2% (38)	54.8% (46)	56.0% (47)	84

**Table 4:** Medical utilization and costs over time (*n* = 269 patients).

	12 months before enrollment	12 months after enrollment
	Mean (SE)
ER visits**	0.84	1.57
ER visit admissions	0.29	0.29
Admits acute	0.41	0.39
Outpatient office visits**	1.42	4.34
Net medical costs	$5847	$5714
Net pharmacy costs**	$3528	$4655
Net medical and pharmacy costs*	$11,676	$13,300

*P < .05, **P < .01.

## References

[1] Thomas MR, Waxmonsky JA, McGinnis GF, Barry CL (2006). Realigning clinical and economic incentives to support depression management within a medicaid population: the Colorado access experience. *Administration and policy in mental health*.

[2] Katon W (2012). Collaborative depression care models: from development to dissemination. *American Journal of Preventive Medicine*.

[3] Thota AB, Sipe TA, Byard GJ (2012). Collaborative care to improve the management of depressive disorders: a community guide systematic review and meta-analysis. *American Journal of Preventative Medicine*.

[4] Katon W, Unützer J, Wells K, Jones L (2010). Collaborative depression care: history, evolution and ways to enhance dissemination and sustainability. *General Hospital Psychiatry*.

[5] Pincus HA, Houtsinger JK, Bachman J, Keyser D (2005). Depression in primary care: bringing behavioral health care into the mainstream. *Health Affairs*.

[6] Dietrich AJ, Oxman TE, Williams JW (2004). Re-engineering systems for the treatment of depression in primary care: cluster randomised controlled trial. *British Medical Journal*.

[7] Wagner EH, Austin BT, Davis C, Hindmarsh M, Schaefer J, Bonomi A (2001). Improving chronic illness care: translating evidence into action. *Health Affairs*.

[8] Von Korff M, Goldberg D (2001). Improving outcomes in depression. *British Medical Journal*.

[9] Gilbody S, Whitty P, Grimshaw J, Thomas R (2003). Educational and organizational interventions to improve the management of depression in primary care: a systematic review. *Journal of the American Medical Association*.

[10] Williams JW, Gerrity M, Holsinger T, Dobscha S, Gaynes B, Dietrich A (2007). Systematic review of multifaceted interventions to improve depression care. *General Hospital Psychiatry*.

[11] Thomas MR, Waxmonsky JA, Gabow PA, Flanders-McGinnis G, Socherman R, Rost K (2005). Prevalence of psychiatric disorders and costs of care among adult enrollees in a medicaid HMO adult population. *Psychiatric Services*.

[12] Kronick R, Gilmer T, Dreyfus T, Lee L (2000). Improving health-based payment for Medicaid beneficiaries: CDPS. *Health Care Financing Review*.

[13] Spitzer RL, Kroenke K, Williams JBW (1999). Validation and utility of a self-report version of PRIME-MD: The PHQ Primary Care Study. *Journal of the American Medical Association*.

[14] Hedeker D, Gibbons R (2006). *Longitudinal Data Analysis*.

[15] Fairclough DL *Design and Analysis of Quality of Life Studies in Clinical Trials*.

[16] Diggle P, Kenward MG (1994). Informative drop-out in longitudinal data analysis. *Applied Statistics*.

[17] Littell RC, Milliken GA, Stroup WW, Wolfinger RD (1996). *SAS System for Mixed Models*.

[18] Kilian R, Matschinger H, Löffler W, Roick C, Angermeyer MC (2002). A comparison of methods to handle skew distributed cost variables in the analysis of the resource consumption in schizophrenia treatment. *Journal of Mental Health Policy and Economics*.

[19] Kroenke K, Spitzer RL, Williams JBW (2001). The PHQ-9: validity of a brief depression severity measure. *Journal of General Internal Medicine*.

[20] Löwe B, Unützer J, Callahan CM, Perkins AJ, Kroenke K (2004). Monitoring depression treatment outcomes with the Patient Health Questionnaire-9. *Medical Care*.

[21] Grypma L, Haverkamp R, Little S, Unützer J (2006). Taking an evidence-based model of depression care from research to practice: making lemonade out of depression. *General Hospital Psychiatry*.

[22] Rost K, Nutting P, Smith J, Werner J, Duan N (2001). Improving depression outcomes in community primary care practice: a randomized trial of the QuEST intervention. *Journal of General Internal Medicine*.

[23] Feldman MD, Areán PA, Ong MK, Lee DL, Feldman S (2005). Incentives for primary care providers to participate in a collaborative care program for depression. *Psychiatric Services*.

[24] Barry CL, Thomas MR (2005). Improving the quality of depression care in medicaid. *Psychiatric Services*.

